# NIPT and the concerns regarding ‘routinisation’

**DOI:** 10.1038/s41431-022-01053-6

**Published:** 2022-02-07

**Authors:** Ruth Horn

**Affiliations:** The Ethox Centre, Nuffield Department of Population Health, University of Oxford, Oxford, UK

**Keywords:** Medical ethics, Health policy

Non-invasive prenatal testing (NIPT) is a rapidly developing technology which is constantly widening its scope in reproductive medicine; a development that is accentuating existing as well as raising new ethical questions [1].

NIPT carries a number of advantages compared to other prenatal tests. This screening test requires less invasive procedures on the woman as it is based on cell-free foetal DNA found in the blood of a pregnant woman and has no associated miscarriage risk. It can be done as early as 9 or 10 weeks of gestation and is more accurate screening for common aneuploidies (e.g. Trisomy (T)21, T18 or T13) than other prenatal tests such as ultrasound scans or the combined first-trimester screening. NIPT for common aneuploidies is not used as a diagnostic test at present and so a positive NIPT test still requires invasive testing (amniocentesis, ChorionicVillus Sampling) for confirmation. However, because of the high accuracy of NIPT, fewer women will be offered confirmatory invasive tests, when they receive a lower risk result, compared to other screening tests. Although there is ongoing development of the test and some commercial companies offer NIPT for a range of other anomalies (e.g. microdeletions or single gene disorders), scientists question the utility of using NIPT for these purposes at present.

Since its introduction in 2011, NIPT has become globally available in the private sector, and increasingly also in the public sector. Whereas some countries (e.g. England, France, Germany) decided to offer NIPT as a fully reimbursed second-tier test (i.e. following initial risk assessment) [2], other countries (e.g. the Netherlands and Belgium) partially reimburse the use of NIPT as a first-tier test.

Despite NIPT’s advantages, offering the test as a public health service raises important ethical questions. One major concern is that NIPT could become routinised, as ‘just another pregnancy test’ and that this risks further undermining reproductive autonomy, a risk that has been associated with genetic prenatal testing for many decades. The concern is that routinisation of NIPT could affect the level of informed choice, increase (social and moral) pressure on women to test, and/or increase the risk of stigmatisation and discrimination of persons affected by a particular condition (e.g. T21).

Van der Meij et al. [3] and Garcia et al. [4] have explored these issues in the Dutch context where NIPT is currently offered as a first-tier screening test as part of a nationwide implementation study (TRIDENT-2). Neither of these studies confirmed the aforementioned concerns about routinization.

Van der Meij et al. conducted a survey with 751 pregnant women who had received counselling prior to prenatal testing. The study indicates that most women made an informed choice in line with their values. Women valued NIPT for its higher accuracy and because it involves only a blood test. Women who took up testing wanted reassurance and further information about their child’s health, and those who declined stated that every child is welcome and would not consider terminating the pregnancy. Most women felt free of any social pressure to accept or decline the test. A very small number of women took up prenatal testing because their partner, other relatives or their healthcare professional wanted it. Women who took up prenatal testing tended to think that it would be a burden to have a child with T21 even though many of them thought that there is good support available in the Netherlands.

Similarly, the qualitative interview study by Garcia et al. conducted with 29 pregnant women, confirmed that women felt no pressure regarding decisions about prenatal testing or other pregnancy decisions. To preserve reproductive autonomy in the context of an affected pregnancy, women emphasised the importance of having support from family and the state. The broader aim of this interview study was to explore whether the availability of a prenatal test that offers high level of accuracy and is based on a simple maternal blood test with no associated miscarriage risk, would increase women’s feeling of moral responsibility to take up NIPT. The results showed that women did not associate the advantages of NIPT with responsible motherhood. The main reason for this was that the test provides only limited information about a small number of fetal conditions that are neither treatable nor preventable (other than through termination of pregnancy). Women argued that there is no duty to take up NIPT as the test does not provide information about the severity or impact of the detected condition (e.g. T21) on the child’s life. Following these results, the authors conclude that pressure on women may increase if and when NIPT is used to screen for treatable or preventable conditions.

Both studies highlight the fact that if information and social support to raise a child with a disability are available, then women find it easy to make decisions about prenatal testing in line with their values and independently of others’ opinions. Informed decision-making and free choice do not appear to be affected because a test can be done easily, is thought to be safer than other tests, or has become part of clinical routine. Women do not perceive a (moral) responsibility or any other social pressure to take a test, particularly if results do not provide clear information about the impact on the health of the child or a variety of courses of action. When providing information and counselling, it is important to clearly communicate the inherent uncertainty of genetic testing in the prenatal context, particularly the fact that testing provides little insight into the types or severity of impairments that an affected child might have. This makes NIPT no different from any other prenatal genetic tests requiring detailed discussion about their scope and limits, as well as about the possible impact of the condition that is tested for, to support reproductive autonomy.
